# Supplementary material for: Particles Morphology Impact on Cytotoxicity, Hemolytic Activity and Sorption Properties of Porous Aluminosilicates of Kaolinite Group
Source: Nanomaterials (Basel). 2022 Jul 26;12(15):2559. doi: 10.3390/nano12152559 (PMC9332423; doi:10.3390/nano12152559)
Supplement: Supplementary file 1 [file nanomaterials-12-02559-s001.zip › nanomaterials-1828950-supplementary.pdf]

*Supporting Information*

# Particles Morphology Impact on Cytotoxicity, Hemolytic Activity and Sorption Properties of Porous Aluminosilicates of Kaolinite Group

**Olga Yu. Golubeva *, Yulia A. Alikina and Elena Yu. Brazovskaya**

Laboratory of silicate sorbents chemistry, Institute of Silicate Chemistry of Russian Academy of Sciences,
Adm.Makarova emb., 2, St. Petersburg 199034, Russia; morozowa_u_a@mail.ru (Y.A.A.); brazovskaya.ics@gmail.com (E.Y.B.)
* Correspondence: olga_isc@mail.ru; Tel.: +7-812-325-21-11

## Summary


| | | |
|---|---|---|
| 1 | **Figure S1.** TEM images of the nanotube samples synthesized at different conditions: a – 350 °C, 72 h, pH 7; b – 220 °C, 72 h, pH 12. | **S2** |
| 2 | **IR spectroscopy studied of the samples with different morphology** | **S3** |
| | **Figure S2.** IR spectra of synthetic aluminosilicates with different morphology and natural nanotubular halloysite. | **S3** |
| | **Table S1.** FT-IR band positions of the samples. | **S4** |
| | **Figure S3.** IR spectra of the samples with spherical (*a*) and nanosponge particles morphology before and after methylene blue (MB) adsorption | **S5** |
| 3 | **Thermal Analysis** | **S6** |
| | **Figure S4.** Differential-thermal and mass spectrometric analysis curves of the samples with different morphology: a – spheres, b – plates, c – nanosponges, d – nanotubes | **S7** |
| | **Figure S5.** Equilibrium adsorption isotherms of methylene blue plotted according to the Langmuir model. | **S8** |

## TEM images of the nanotube samples

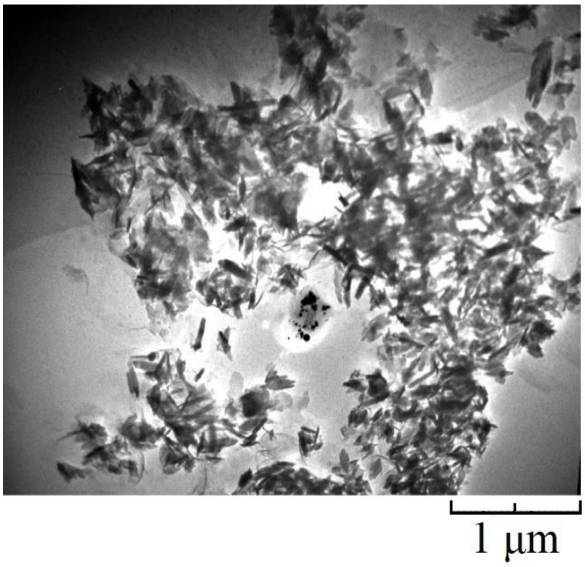


*a*

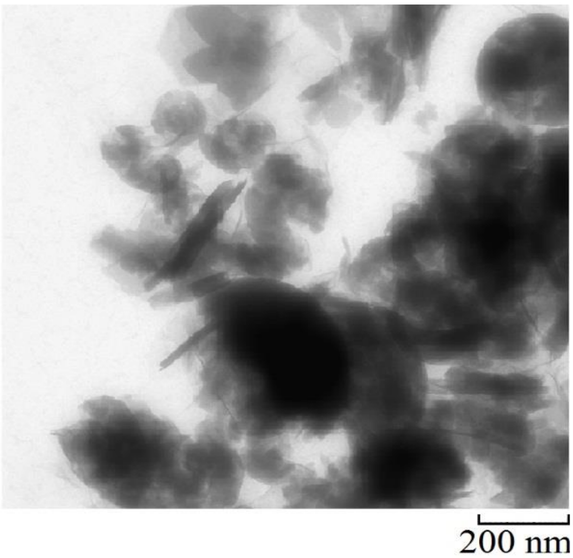


*b*

**Figure S1**. TEM images of the nanotube samples synthesized at different conditions: a – 350 °C, 72 h, ph 7; b – 220 °C, 72 h, pH 12.

Figure S1 shows images of the studied samples with the kaolinite structure containing particles with nanotubular morphology. This proves that nanotubes can be formed in hydrothermal conditions; however, nanotube morphology is not the main one for any of the samples.

## IR spectroscopy studied of the samples with different morphology

Analysis of the structure of the samples and the presence of new chemical bonds were carried out by infrared (IR) spectroscopy using a Fourier Transform Infrared Spectrophotometer Shimadzu IRTracer-100 at room temperature (Shimadzu Corporation, Kyoto, Japan). All spectra were collected over 50 scans in the range from 4000 to 350 cm$^{-1}$ at a resolution of 4 cm$^{-1}$. Samples for measurements were made by pressing the studied powders with KBr under vacuum. The concentration of the sample in KBr was 1 wt %. To remove water, KBr and the initial samples were preliminarily heated at 120 °C for 24 h.

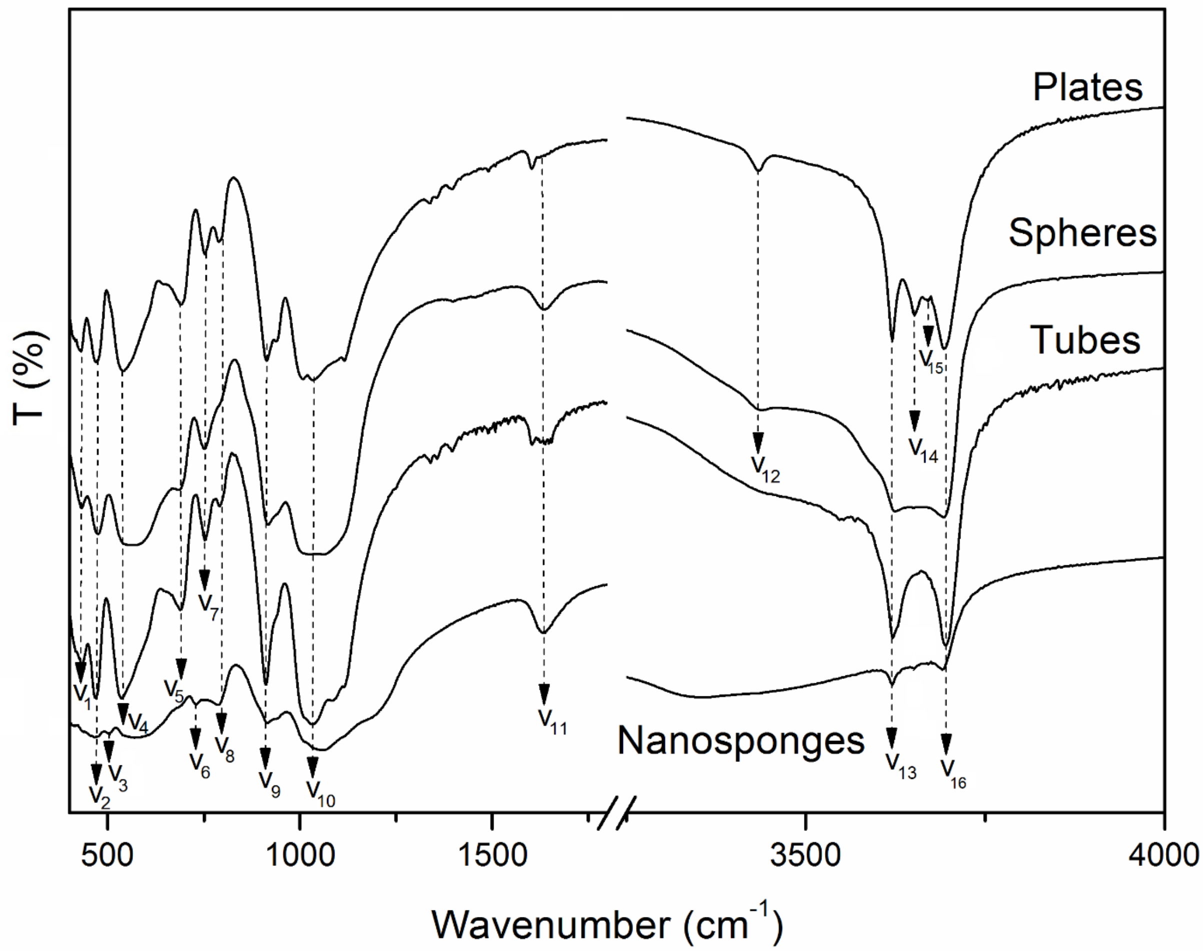


**Figure S2**. IR spectra of synthetic aluminosilicates with different morphology and natural nanotubular halloysite.

Figure S2 shows the IR spectra of natural nanotubular halloysite and synthetic aluminosilicates of various morphologies. The range of stretching vibrations of the OH groups of the lamellar sample is characterized by four bands at 3695, 3669, 3652, and 3620 cm$^{-1}$, which indicates a high degree of order. The band at 3620 cm$^{-1}$ arises from the stretching vibrations of single "internal" hydroxyl groups ($\nu(OH_{in})$) associated with octahedral cations but located mainly

in the plane of the apical oxygen atoms that link the tetrahedral and octahedral networks. The remaining three bands are due to coupled stretching vibrations of nonequivalent “internal” OH groups located on the surface of the dioctahedral network.

Although the chemical composition of natural nanotubular halloysite, spherical and nanosponge samples is similar to lamellar aluminosilicate, their IR spectra show only two bands at 3695 $cm^{-1}$ and 3620 $cm^{-1}$ in the region of OH stretching vibrations. The band at 1630 and 3435 $cm^{-1}$ refers to adsorbed water. The band in the range 912-950 $cm^{-1}$ arises due to Al2OH vibrations, and 1034 $cm^{-1}$ due to Si-O stretching vibrations. The bands at 537, 503, and 430 $cm^{-1}$ are due to deformation vibrations of Al-O-Si, Si – O – Si, and Si – O, respectively, and their intensity corresponds to the degree of ordering of kaolinite. The rest of the characteristic bands and their values are given in table S1.

**Table S1**. FT-IR band positions of the samples.

| band | Transmission, $cm^{-1}$ | Group |
|---|---|---|
| $v_1$ | 430 | δ(Si-O) |
| $v_2$ | 467 | δ(Si-O-Si) |
| $v_3$ | 503 | δ(Si-O-Si) |
| $v_4$ | 537 | δ(Al-O-Si) |
| $v_5$ | 691 | δ(Si-O) |
| $v_6$ | 729 | δ(Si-O) |
| $v_7$ | 752 | δ(Si-O) |
| $v_8$ | 791 | δ(Si-O) |
| $v_9$ | 912 | $\delta(OH_{in})$ |
| $v_{10}$ | 1034 | ν(Si-O) |
| $v_{11}$ | 1635 | H-O-H |
| $v_{12}$ | 3433 | H-O-H |
| $v_{13}$ | 3620 | $\nu(OH_{in})$ |
| $v_{14}$ | 3652 | $\nu\ (OH_{in-s})$ |
| $v_{15}$ | 3669 | $\nu(OH_{in})$ |
| $v_{16}$ | 3695 | $\nu(OH_{in-s})$ |

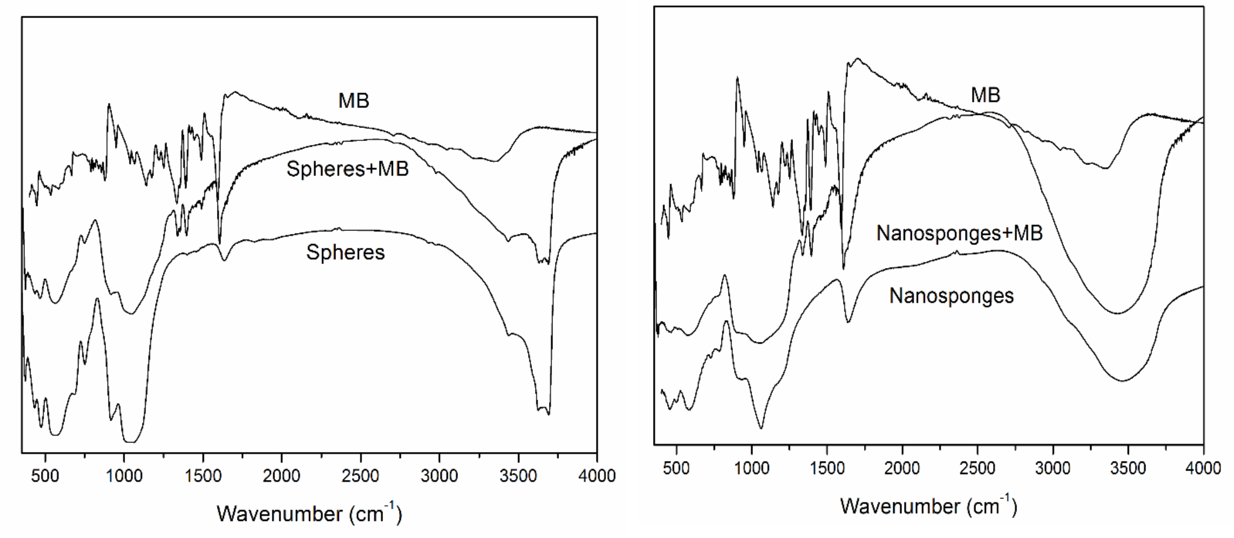


*a* *b*

**Figure S3.** IR spectra of the samples with spherical (a) and nanosponge (*b*) particles morphology before and after methylene blue (MB) adsorption.

It can be seen in Figure S3 that after the adsorption of methylene blue, the formation of new chemical bonds is not fixed, therefore it cannot be concluded that chemisorption takes place.

**Thermal analysis**

The experiments were carried out on an STA 429 CD device for synchronous thermal analysis of the German company NETZSCH using a platinum-platinum-rhodium holder for samples of the "TG + DSC" type. To analyze the decomposition products, a QMS 403 C quadrupole mass spectrometer of the same company was used, which makes it possible to analyze thermal decomposition products in the range from 1 to 121 atomic-charge units.

During the analysis, the changes in the sample mass in % of the sample weight (TG curve) and the enthalpy changes accompanying thermal transformations, in microwatts per milligram (DSC curve), as well as the curves of changes in the values of ion currents (IC curves) caused by different ion masses in thermal decomposition products were determined.

For research, tablets with a diameter of 5.05 mm and a thickness of about 0.8-0.9 mm were pressed from the powders, the pressing pressure was about 1 kg / $mm^2$, the mass of the tablets was about 20 mg. After weighing on an analytical balance with an accuracy of ± 0.01 mg, the tablets were placed in an alundum crucible without a lid and placed on a holder, and then subjected to complex thermal analysis when heated at a rate of 20 °C per minute in a dynamic air atmosphere (flow 50 $cm^3$ in minute) in the range from 40 to 1100 °C.

Figure S3 shows the TG, DSC and IC curves (ion current due to ions with a mass of 18) obtained by heating a pellet of aluminosilicate samples in the range from 40 to 1100 °C at a rate of 20 °C per minute in a dynamic air atmosphere. The results of differential thermal analysis of the samples are shown in Fig. S3. TG and DSC curves for raw halloysite (Fig. S3 *d*) show a typical picture of its thermal decomposition. At temperatures of 150-370°C, a mass loss of 0.8 wt.% occurs, associated with the removal of physically absorbed water, and an endothermic peak at 518°C with a loss of 10 wt.% - with the release of structurally bound water, which is accompanied by a peak on the ionic current corresponding to mass 18. An intense exothermic reaction at 1000°C is observed reflecting the crystallization of mullite. The platy morphology sample (Fig. S3 *b*) exhibits the same thermal effects. For a sample with a spherical morphology (Fig. S3 *a*), the removal of the physically bound material is accompanied by greater mass losses (up to 10 wt %) than for samples with tubular and lamellar morphologies. For a sample with spherical morphology (Fig. S3 *a*), the removal of physically bound water is accompanied by greater mass losses (up to 10 wt %) than for samples with tubular and lamellar morphologies. At the same time, there is less structurally bound water in the spherical sample, as evidenced by the endothermic peak at 518 °C with a loss of 2.6 wt.%. Even more physically absorbed water is contained in the sample of nanosponges - while the endothermic peak in the region of 500 °C is not observed, which indicates the almost complete absence of structurally bound water. Such differences in the thermal behavior of the samples and in the content of various forms of water in them can be associated with

differences in their specific surface area and porosity. In particular, a larger pore volume in samples with spherical and sponge morphologies leads to a higher content of physically absorbed water in them.

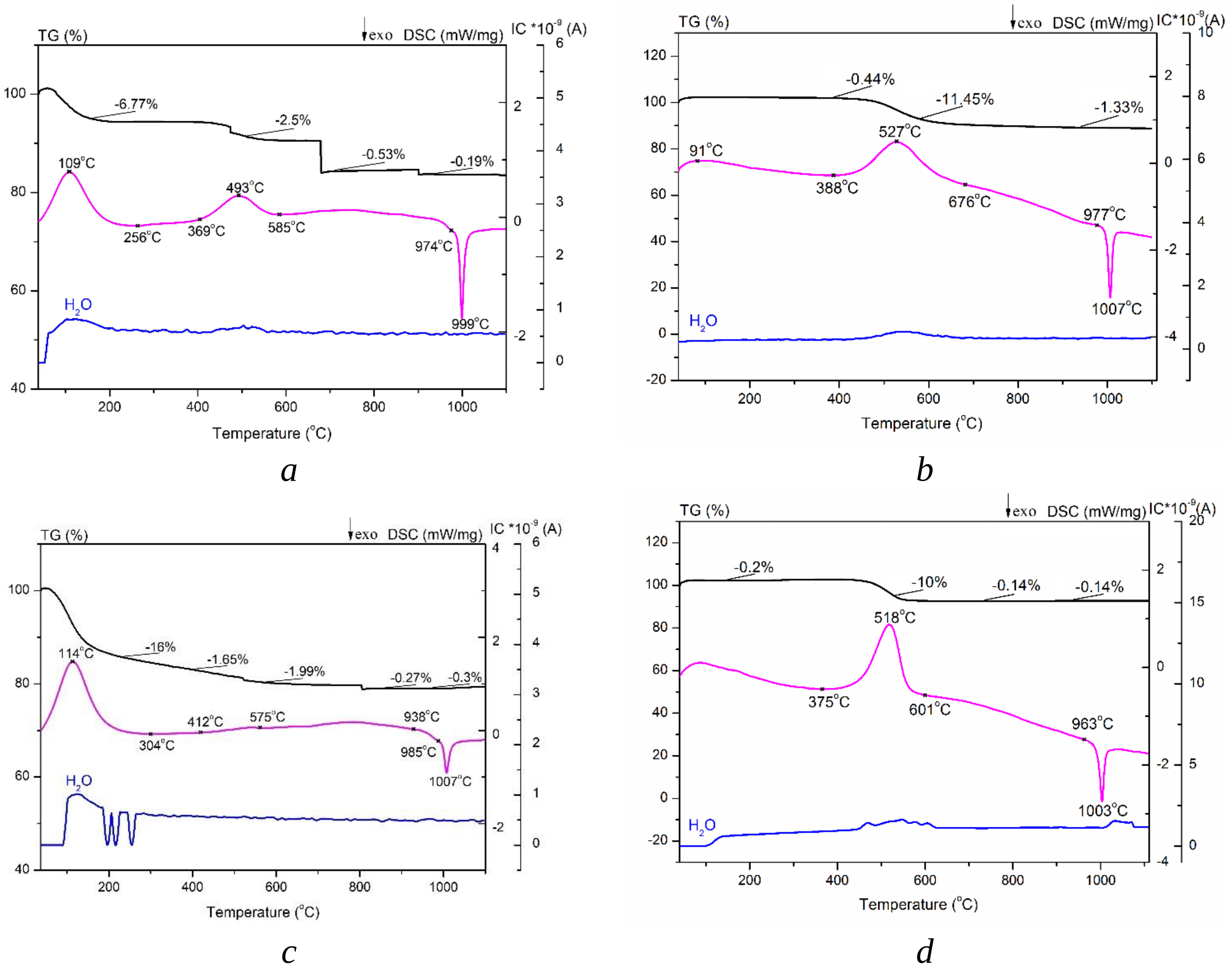


**Figure S4**. Differential-thermal and mass spectrometric analysis curves of the samples with different morphology: *a* – spheres, *b* – plates, *c* – nanosponges, *d* – nanotubes.

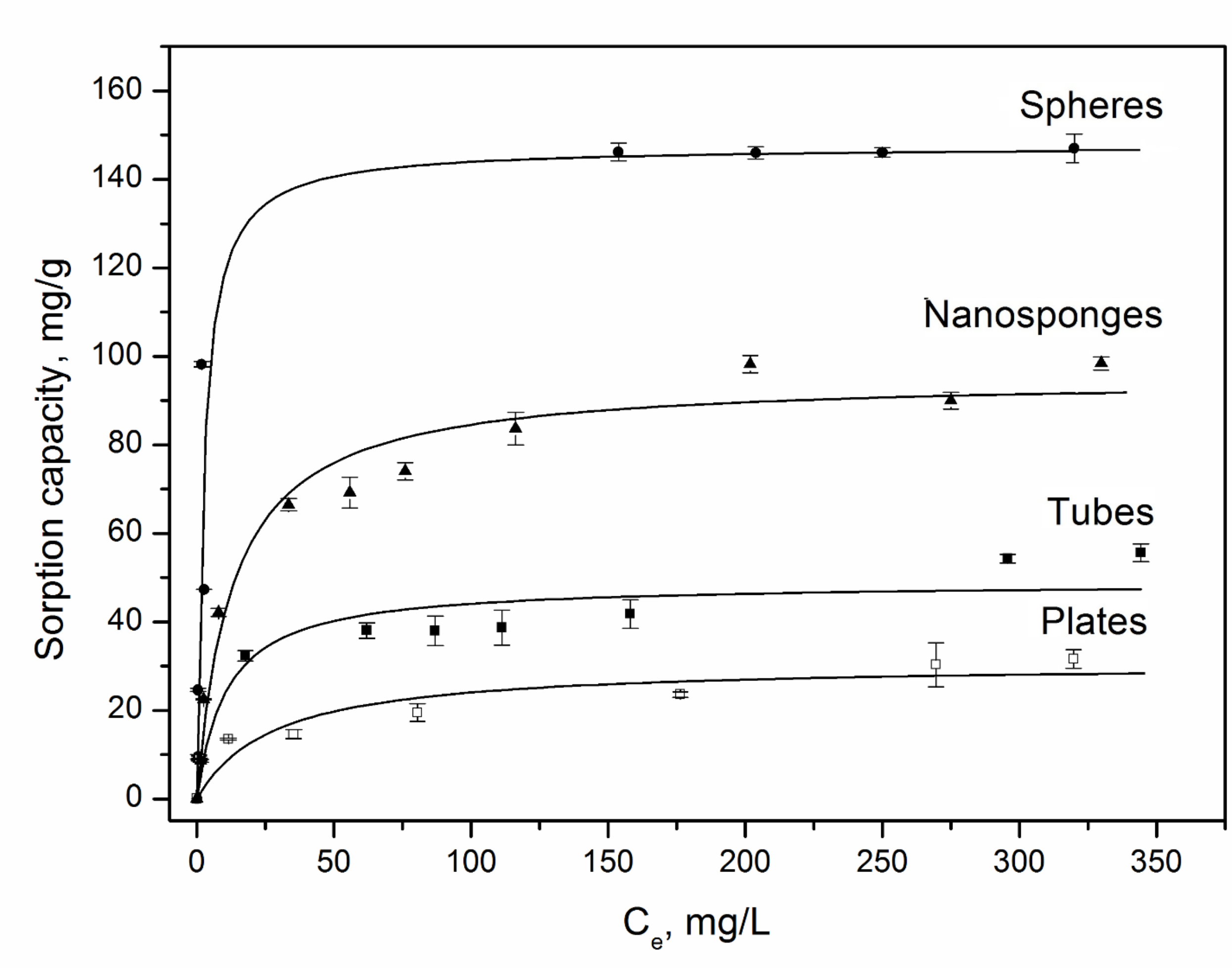


**Figure S5**. Equilibrium adsorption isotherms of methylene blue plotted according to the Langmuir model
